# Macrophages with different origins proliferate *ex vivo* and do not lose their core intrinsic features

**DOI:** 10.1016/j.isci.2025.112635

**Published:** 2025-05-12

**Authors:** Sara A. Habash, Naofumi Takahashi, Youssef M. Eltalkhawy, Randa A. Abdelnaser, Hiromi Ogata-Aoki, Seiji Okada, Hitoshi Takizawa, Shingo Usuki, Kan Etoh, Shinjiro Hino, Saori Morino-Koga, Minetaro Ogawa, Shinya Suzu

**Affiliations:** 1 Division of Infection & Hematopoiesis, Kumamoto University, Kumamoto, Japan; 2 Division of Hematopoiesis, Joint Research Center for Human Retrovirus Infection, Kumamoto University, Kumamoto, Japan; 3 Laboratory of Stem Cell Stress, International Research Center for Medical Sciences (IRCMS), Kumamoto University, Kumamoto, Japan; 4 Liaison Laboratory Research Promotion Center, Kumamoto University, Kumamoto, Japan; 5 Department of Medical Cell Biology, Kumamoto University, Kumamoto, Japan; 6 Department of Cell Differentiation, Institute of Molecular Embryology and Genetics, Kumamoto University, Kumamoto, Japan; 7 Clinical Pathology, Faculty of Medicine, Suez Canal University, Ismailia, Egypt; 8 Department of Refractory Viral Infections, National Center for Global Health & Medicine Research Institute, Tokyo, Japan

**Keywords:** Biological sciences, Immunology, Components of the immune system

## Abstract

Macrophages are maintained by bone marrow (BM)–derived monocytes, but macrophages originating from fetal liver (FL) or yolk sac (YS) persist in many adult tissues due to their higher proliferative capacity. Here, we report useful models of macrophages with different origins. We expanded macrophages from mouse BM, FL, or YS through long-term culture. They proliferated with M-CSF, and YS lines were the fastest and survived without M-CSF for a longer period. YS and FL lines were more resistant to apoptotic cell death and similar in gene expression/chromatin accessibility, whereas YS and BM lines exhibited the most distinct profiles. When transplanted into mice, YS line expressed markers lost during the culture. BM, FL, and YS lines appear to maintain differences in intrinsic proliferation/anti-apoptosis/survival capacities and can restore phenotypes lost during the culture by *in vivo* transfer. Our models help in the understanding of physiological/pathological roles of macrophages with different origins.

## Highlights

• Self-renewing macrophages can be expanded from yolk sac, fetal liver, or bone marrow
• Macrophages from yolk sac and fetal liver are relatively close in their phenotypes
• Yolk sac–derived macrophages are the most proliferative and resistant to apoptosis
• When transplanted into mice, yolk sac–derived macrophages survive and proliferate

## Introduction

Tissue macrophages are specialized innate immune cells that orchestrate homeostasis, inflammation, and regeneration,^1^ and have been thought to be maintained by a constant replenishment through the differentiation of bone marrow (BM)–derived peripheral blood monocytes.^2^ However, recent studies revealed that macrophages originating from yolk sac (YS) or fetal liver (FL) persist in many adult tissues.^3^^,^^4^^,^^5^ This paradigm shift in our understanding of macrophage development raises many questions, such as, how the precursors in YS, monocytes or erythro-myeloid progenitors^6^^,^^7^ in FL, and BM-derived monocytes contribute to maintaining macrophage pool in each tissue, and how these macrophages with different origins are phenotypically and functionally different from each other.

In general, macrophages originating from YS or FL are thought to have a high and long-term proliferative capacity, which may explain why those embryonic macrophages persist in adult tissues.^3^^,^^4^^,^^5^ In fact, it was reported that the precursors in YS and monocytes in FL, but not monocytes from BM, shared an enriched expression of cell proliferation–associated genes.^8^ Consistent with this, Fejer et al. reported an *ex vivo* expansion of mouse FL -derived macrophages, which could proliferate *in vitro* for an extended period in almost unlimited numbers in the presence of a cytokine GM-CSF.^9^

Meanwhile, several *in vivo* studies revealed that BM-derived monocytes also differentiate into proliferative macrophages under certain circumstances.^10^^,^^11^^,^^12^^,^^13^ Consistent with this, Ito et al. reported the *ex vivo* expansion of mouse BM-derived macrophages using GM-CSF.^14^ Interestingly, macrophages with BM origin proliferated in the Zymosan-induced peritonitis mouse model, in a manner dependent on M-CSF.^10^ M-CSF (also known as CSF-1) is the cytokine required for macrophage development in many tissues.^15^^,^^16^ In fact, we recently demonstrated that macrophages could be expanded by long-term culture of BM cells with M-CSF, and that they were non-tumorigenic but stably proliferated in unlimited numbers in the presence of M-CSF,^17^ as with GM-CSF-expanded FL or BM-derived macrophages.^9^^,^^14^

The findings mentioned above suggest that tissue macrophages can be maintained by the replenishment through the differentiation of BM-derived monocytes and the proliferation of macrophages originating from BM, FL, or YS.^18^^,^^19^^,^^20^ Those findings also suggest that macrophages originating from FL or YS are more proliferative than macrophages originating from BM.^18^^,^^19^^,^^20^ To prove the idea, the *ex vivo*-expanded stably proliferating macrophages are helpful as they are untransformed and genetically unmodified.^9^^,^^14^^,^^17^ However, different groups expanded macrophages from fetal liver^9^ or BM,^14^^,^^17^ using different cytokines (GM-CSF or M-CSF), which makes it difficult to precisely compare their phenotypes. Moreover, it remains unexplored whether macrophages expanded from YS can proliferate in unlimited numbers,^21^ as with macrophages expanded from fetal liver or BM.^9^^,^^14^^,^^17^

In this study, we successfully expanded macrophages from BM, fetal liver, and YS under the same culture conditions in unlimited numbers, and compared their phenotypes under the same setting. Here, we report that the expanded macrophages do not entirely lose their core intrinsic features including the proliferation even after long-term culture with repeated passages.

## Results

### Macrophages expanded from bone marrow (BM), fetal liver (FL), or yolk sac (YS) are phenotypically and functionally overlapping but not identical

In the previous study, we cultured BM cells with M-CSF, repeated the detachment/reseeding when macrophages proliferated, and obtained stably proliferating BM-derived macrophages.^17^ In this study, by employing the same methods, we obtained stably proliferating macrophages from FL or YS (Figure S1). They could be maintained in continuous culture for at least 18 months after the expansion in the presence of M-CSF, and could be frozen without losing the proliferative capacity. The bulk culture obtained from a separate dish or well was defined as an independent line. They were hereafter referred to as BM lines, FL lines, or YS lines. We used these lines with a similar number of passages for each experiment.

**Figure 1:**
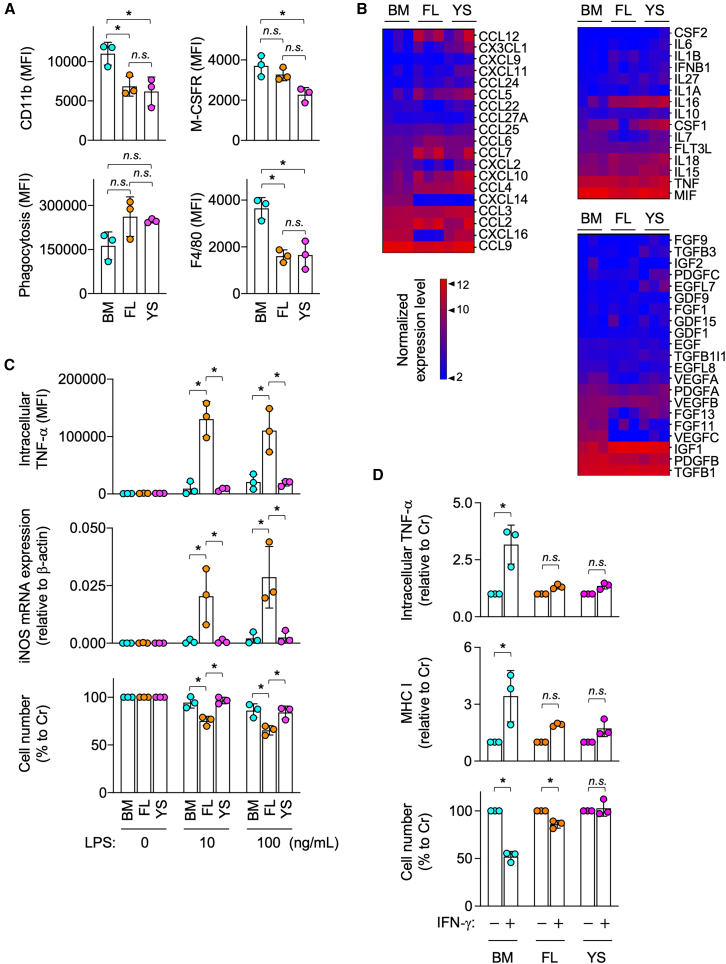
Expression of cell surface molecules or chemokines/cytokines/growth factors, phagocytic activity, and response to LPS or IFN-γ of expanded macrophages (A) BM, FL, or YS lines (*n* = 3 for each group) were analyzed for the cell surface expression of CD11b, M-CSF receptor (M-CSFR) or F4/80 by flow cytometry. They were also analyzed for the phagocytic activity (the uptake of fluorescent microspheres) by flow cytometry. The mean fluorescence intensity (MFI) is shown (mean ± SD). *n.s.*, not significant. ^∗^*p* < 0.05. [One-way ANOVA]. (B) BM, FL, or YS lines (*n* = 3 for each group) were subjected to bulk RNA-seq analysis. The indicated chemokines (upper left), cytokines (upper right), and growth factors (lower right) were selected, and their normalized expression levels are summarized in the heat maps. The color scale is shown (lower left). (C) BM, FL, or YS lines (*n* = 3 for each group) were cultured in the absence or presence of 10 or 100 ng/mL of LPS for 8 h, and analyzed for the intracellular level of TNF-α protein by flow cytometry. The MFI is shown (top). They were also cultured in the absence or presence of LPS for 6 h, and analyzed for the expression of iNOS mRNA by qRT-PCR. The expression level of iNOS mRNA relative to that of β-actin mRNA is shown (middle). They were also cultured in the absence or presence of LPS for 24 h, and their survival was assessed by the MTT assay. The level shown is the percentage to that of the LPS-free control culture (bottom) (mean ± SD). *n.s.*, not significant. ^∗^*p* < 0.05. [two-way ANOVA with Tukey’s multiple comparisons test]. (D) BM, FL, or YS lines (*n* = 3 for each group) were cultured in the absence or presence of 10 ng/mL IFN-γ for 24 h, and analyzed for the intracellular level of TNF-α protein or cell surface level of MHC I (H-2) by flow cytometry (top and middle). The level shown is relative to that of the IFN-γ-free control culture. Also, their survival was assessed by the MTT assay. The level shown is the percentage to that of the IFN-γ-free control culture (bottom) (mean ± SD). *n.s.*, not significant. ^∗^*p* < 0.05. [two-way ANOVA with Sidak’s (top and middle) or Tukey’s (bottom) multiple comparisons test] See also Figures S1–S3.

All the lines were positive for the myeloid marker CD11b, the macrophage markers M-CSF receptor (M-CSFR) and F4/80, and the hallmark macrophage function phagocytic activity (Figures 1A and S2). They also showed the relatively similar expression profile of chemokines, cytokines, or growth factors (Figure 1B). For example, the normalized expression levels of M-CSF (CSF1) of BM, FL, and YS lines were 6.48 ± 1.26, 5.73± 2.46 and 8.96± 0.63, respectively (Figure 1B, upper right). Meanwhile, FL lines produced TNF-α protein and expressed inducible nitric oxide synthase (iNOS) mRNA in response to bacterial lipopolysaccharide (LPS) at much higher levels than BM or YS lines (Figure 1C, top and middle panels). FL lines also showed activation-induced cell death in response to LPS more prominently than BM or YS lines (Figure 1C, bottom panel). Such strong response to LPS was reported for unexpanded monocytes in FL.^22^ In contrast, BM lines responded to IFN-γ more strongly than FL or YS lines: BM lines produced TNF-α or expressed MHC I in response to IFN-γ at higher level than FL or YS lines (Figure 1D, top and middle panels). FL and YS lines were responsive to IFN-γ, albeit weakly (Figure S3). Consistent with this, BM lines showed the most obvious activation-induced cell death in response to IFN-γ (Figure 1D, bottom panel). These results indicated that BM, FL, and YS lines were overlapping but not identical in phenotypes and functions, despite long-term culture with repeated passages.

### FL and YS lines are relatively close in gene expression profile and chromatin accessibility, but distant from BM lines

**Figure 2:**
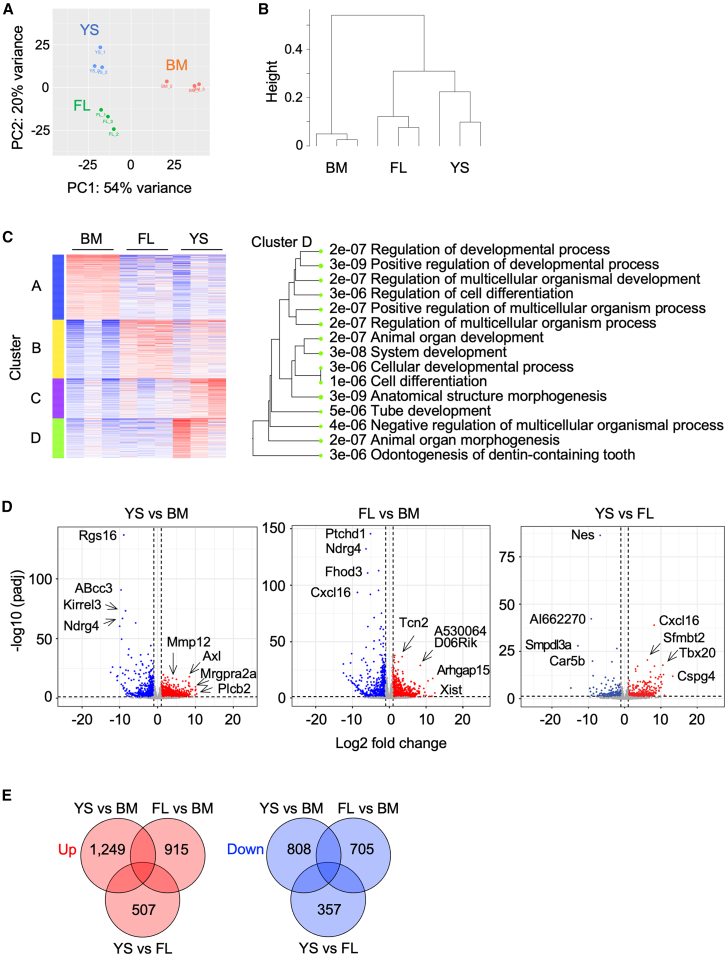
Gene expression profile of expanded macrophages (A–E) BM, FL, or YS lines (*n* = 3 for each group) were subjected to bulk RNA-seq analysis. (A) Principal component analysis of BM lines (orange), FL lines (green), and YS lines (blue). (B) Dendrogram showing the hierarchical clustering of BM, FL, and YS lines. (C) The most variable 2,000 genes among three groups categorized using *k*-means clustering (k = 4) are summarized in the heatmap (left). The gene ontology terms for biological process enriched in YS lines (Cluster D) in the left heatmap are also shown (right). (D) Volcano plots showing differentially expressed genes in all pairwise comparisons are summarized (fold change >2 and false discovery rate <0.1). padj, adjusted *p*-value. (E) The numbers of differentially expressed genes in all pairwise comparisons are summarized (fold change >2 and false discovery rate <0.1). The numbers of upregulated genes (left) or downregulated genes (right) are shown. See also Figures S4 and S5.

We next compared the gene expression profile of expanded macrophages using RNA-seq. The principal component analysis (Figure 2A), the dendrogram (Figure 2B), and the heatmap of top 50 differentially expressed genes (Figure S4) suggested that FL and YS lines were relatively close, but distant from BM lines. When the most variable 2,000 genes were categorized using *k*-means clustering (Figures 2C and S5), genes involved in developmental process, multicellular organism process, or organ development or morphogenesis were enriched in YS lines (Figure 2C, Cluster D), suggesting that YS lines retain some degree of their original features. When differentially expressed genes were shown in volcano plots (Figure 2D) or Venn diagrams (Figure 2E), the difference between YS and FL lines was smaller than that between YS and BM lines or FL and BM lines. For example, 2,057 (1,249 upregulated +808 downregulated), 1,620 (915 upregulated +705 downregulated), and 864 (507 upregulated +357 downregulated) genes were differentially expressed between YS and BM lines, FL and BM lines, and YS and FL lines, respectively (Figure 2E). These results suggested that FL and YS lines were relatively close, but distant from BM lines in the gene expression profile.

**Figure 3:**
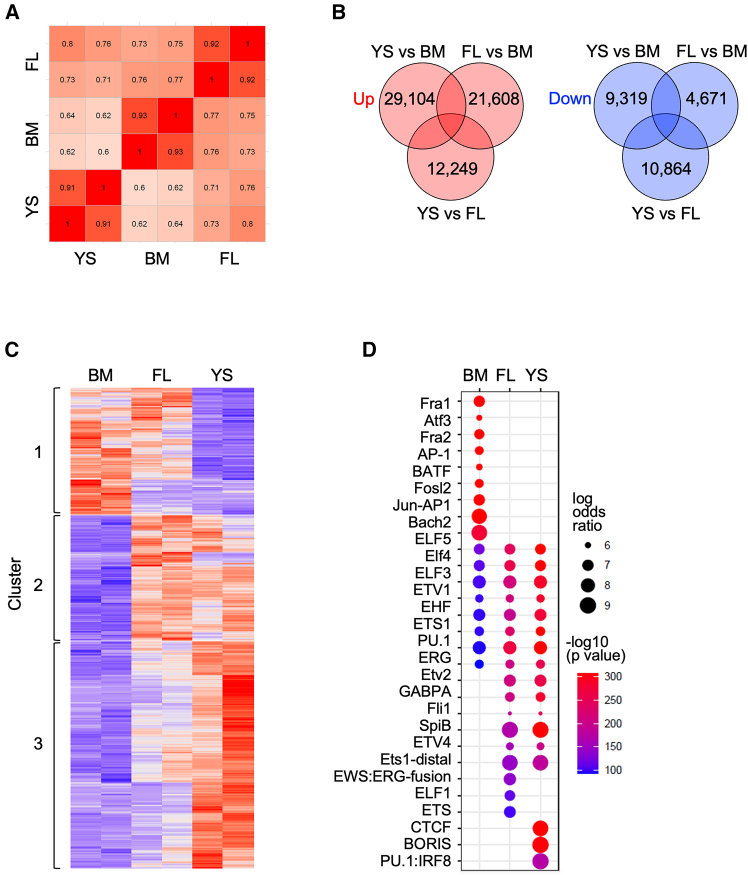
Chromatin accessibility of expanded macrophages (A–D) BM, FL, or YS lines (*n* = 2 for each group) were subjected to ATAC-Seq analysis. (A) Pearson’s correlation coefficients of all pairwise comparisons are shown in the heatmap. (B) The numbers of differentially accessible peaks in all pairwise comparisons are summarized (fold change >2 and false discovery rate <0.1). The numbers of upregulated peaks (left) or downregulated peaks (right) are also shown. (C and D) In C, the most variable 7,024 peaks among three groups are summarized in the heatmap using *k*-means clustering (k = 3). In D, the transcription factor binding motifs enriched in the clusters in C are summarized in the dot plot. The dot color and size represent -log10 (*p* value) and log odds ratio, respectively. See also Figure S6.

We also compared the chromatin accessibility of expanded macrophages using ATAC-seq. Recently, Dick et al. identified the core cell surface markers of embryo-derived macrophages, that is, phosphatidylserine receptor T cell immunoglobulin and mucin domain containing 4 (TIMD4), lymphatic vessel endothelial hyaluronan receptor 1 (LYVE1), and folate receptor beta (FOLR2).^23^ Consistent with this, the open chromatin regions in TIMD4, LYVE1, and FOLR2 of BM lines were different from those of YS lines (Figure S6). The Pearson’s correlation coefficients (Figure 3A) suggested that FL and YS lines were relatively similar in the chromatin accessibility whereas BM and YS lines exhibited the most distinct profiles. In fact, as summarized in Figure 3B, 38,423 (29,104 upregulated +9,319 downregulated), 26,279 (21,608 upregulated +4,671 downregulated), and 23,113 (12,249 upregulated +10,864 downregulated) peaks were differentially accessible between YS and BM lines, FL and BM lines, and YS and FL lines, respectively. The heatmap (Figure 3C) that summarized the most variable 7,024 peaks among BM, FL, and YS lines, and the dot plot (Figure 3D) that summarized the transcription factor binding motifs enriched in the clusters in Figure 3C further supported the idea that FL and YS lines were relatively close, but distant from BM lines in the chromatin accessibility.

### YS lines are more proliferative than FL or BM lines

**Figure 4:**
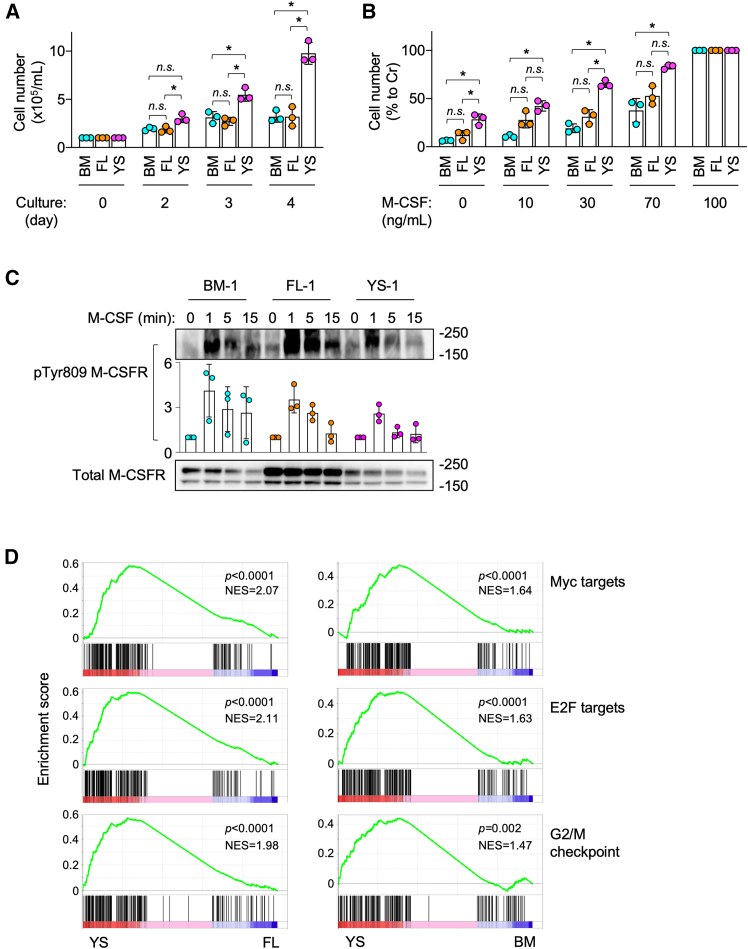
Proliferation of expanded macrophages (A) BM, FL, or YS lines (*n* = 3 for each group) were seeded at 1 × 10^5^ cells/mL and cultured in the presence of 100 ng/mL M-CSF, and the number of viable cells was counted on day 2, 3, or 4, by trypan blue dye exclusion method (mean ± SD). *n.s.*, not significant. ^∗^*p* < 0.05. [two-way ANOVA with Tukey’s multiple comparisons test]. (B) BM, FL, or YS lines (*n* = 3 for each group) were seeded at 1 × 10^5^ cells/mL and cultured for 3 days in the absence or presence of the indicated concentrations of M-CSF (10, 30, or 70 ng/mL), and the number of cells was monitored by the MTT assay. The number of cells shown is the percentage to that of the culture containing 100 ng/mL M-CSF (mean ± SD). *n.s.*, not significant. ^∗^*p* < 0.05. [two-way ANOVA with Tukey’s multiple comparisons test]. (C) BM line #1, FL line #1, or YS line #1 was M-CSF-depleted for 3 h, left untreated or treated with 100 ng/mL M-CSF for the indicated periods (1, 5, or 15 min), and analyzed for the phosphorylated (=activated) M-CSF receptor by western blotting using antibody specific for tyrosine-809-phosphorylated M-CSF receptor^24^ (pTyr809 M-CSFR). Total M-CSF receptor (Total M-CSFR) was also analyzed as a reference. The images shown are representative of three independent experiments. The band density of pTyr809 M-CSFR was normalized to that of total M-CSFR, and the degree of phosphorylated M-CSF receptor shown is relative to that of untreated control cells (*n* = 3) (mean ± SD). (D) BM, FL, or YS lines (*n* = 3 for each group) were subjected to the gene set enrichment analysis of cell proliferation-related gene sets (Myc targets, E2F targets, and G2/M checkpoint), using RNA-seq data. The normalized enrichment score (NES) and *p* value are summarized. YS versus FL and YS versus BM are shown in left and right panels, respectively. See also Figures S7 and S8.

We next compared the proliferative capacity of expanded macrophages. Although all the lines proliferated in the presence of the optimal concentration of M-CSF (100 ng/mL), YS lines showed the fastest proliferation (Figure 4A). The doubling times of BM, FL, and YS lines were 2.97 ± 0.73, 2.66± 0.58, and 2.15± 0.32 days, respectively (Figure S7). The fastest proliferation of YS lines was the case at suboptimal concentrations of M-CSF (10, 30, or 70 ng/mL) (Figures 4B and S8). Under the suboptimal conditions, FL lines tended to proliferate faster than BM lines, although the difference was not significant (Figure 4B). The high proliferative capacity of YS lines in the presence of M-CSF was not necessarily related to the degree of signal activation because the YS line had the smallest amount of phosphorylated M-CSF receptor at Tyr809 (=activated)^24^ upon M-CSF-stimulation, among BM, FL, and YS lines (Figure 4C). Instead, genes of proliferation-related pathways, such as Myc targets, E2F targets, and G2/M checkpoint, were enriched in YS lines (Figure 4D). These results indicated that YS lines were more proliferative than BM or FL lines, which was likely due to the difference in their intrinsic gene expression profiles.

### YS and FL lines are more resistant to apoptosis than BM lines

**Figure 5:**
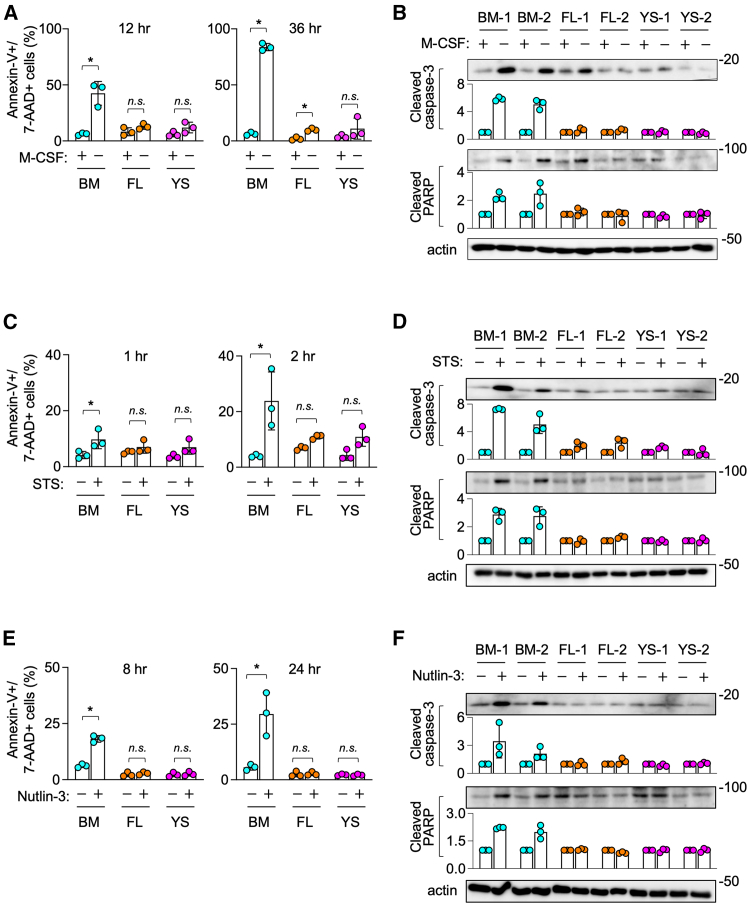
Apoptosis of expanded macrophages (A) BM, FL, or YS lines (*n* = 3 for each group) were cultured with (+) or without (−) M-CSF for 12 h (left) or 36 h (right), and analyzed for the percentage of cells positive for annexin-V and 7-AAD by flow cytometry (mean ± SD). *n.s.*, not significant. ^∗^*p* < 0.05. [two-way ANOVA with Sidak’s multiple comparisons test]. (B) BM, FL, or YS lines (*n* = 2 for each group) were cultured with (+) or without (−) M-CSF for 6 h, and analyzed for cleaved caspase-3 or cleaved PARP by western blotting. β-actin blot is the loading control. The images shown are representative of three independent experiments. The graphs were prepared by quantifying the band density using those three independent experiments (mean ± SD). The level shown is relative to that of M-CSF-containing culture. (C) BM, FL, or YS lines (*n* = 3 for each group) were seeded with M-CSF, cultured for 1 h (left) or 2 h (right) in the absence or presence of staurosporine (STS), and analyzed as in A (mean ± SD). *n.s.*, not significant. ^∗^*p* < 0.05. [two-way ANOVA with Sidak’s multiple comparisons test]. (D) BM, FL, or YS lines (*n* = 2 for each group) were seeded with M-CSF, cultured for 1 h in the absence or presence of STS, and analyzed as in B. The images shown are representative of three independent experiments. The graphs were prepared by quantifying the band density using those three independent experiments (mean ± SD). The level shown is relative to that of the STS-free control culture. (E) BM, FL, or YS lines (*n* = 3 for each group) were seeded with M-CSF, cultured for 8 h (left) or 24 h (right) in the absence or presence of nutlin-3, and analyzed as in A (mean ± SD). *n.s.*, not significant. ^∗^*p* < 0.05. [two-way ANOVA with Sidak’s multiple comparisons test]. (F) BM, FL, or YS lines (*n* = 2 for each group) were seeded with M-CSF, cultured for 5 h in the absence or presence of nutlin-3, and analyzed as in B. The images shown are representative of three independent experiments. The graphs were prepared by quantifying the band density using those three independent experiments (mean ± SD). The level shown is relative to that of the nutlin-3-free control culture.

In the proliferation assay, the number of YS lines that survived without M-CSF for 3 days was higher than that of FL or BM lines (see Figure 4B, M-CSF “0”), suggesting that YS lines are relatively resistant to cell death. Thus, we next compared the cell death or survival of expanded macrophages in more detail. As early as 12 h after M-CSF-depletion, the apoptotic cells positive for both annexin V and 7-AAD were detected for BM lines (Figure 5A, left panel). Such apoptotic cells were few for FL or YS lines, even at 36 h after the M-CSF-depletion (Figure 5A, right panel). Consistent with this, the cleaved caspase-3 and cleaved poly ADP-ribose polymerase (PARP) were detected as early as 6 h after the M-CSF-depletion in BM lines, but not in FL or YS lines (Figure 5B).

The widely used apoptosis-trigger staurosporine (STS) induced the apoptotic cells (Figure 5C) and the cleavage of caspase-3 or PARP (Figure 5D), most obviously in BM lines. A similar result was obtained for another apoptosis-trigger nutlin-3, the inhibitor of p53 – MDM2 interaction^25^ (Figures 5E and 5F). These results indicated that YS and FL lines were more resistant to apoptosis than BM lines, under at least three different conditions.

### YS lines survive for a longer period than BM or FL lines under M-CSF-free culture

**Figure 6:**
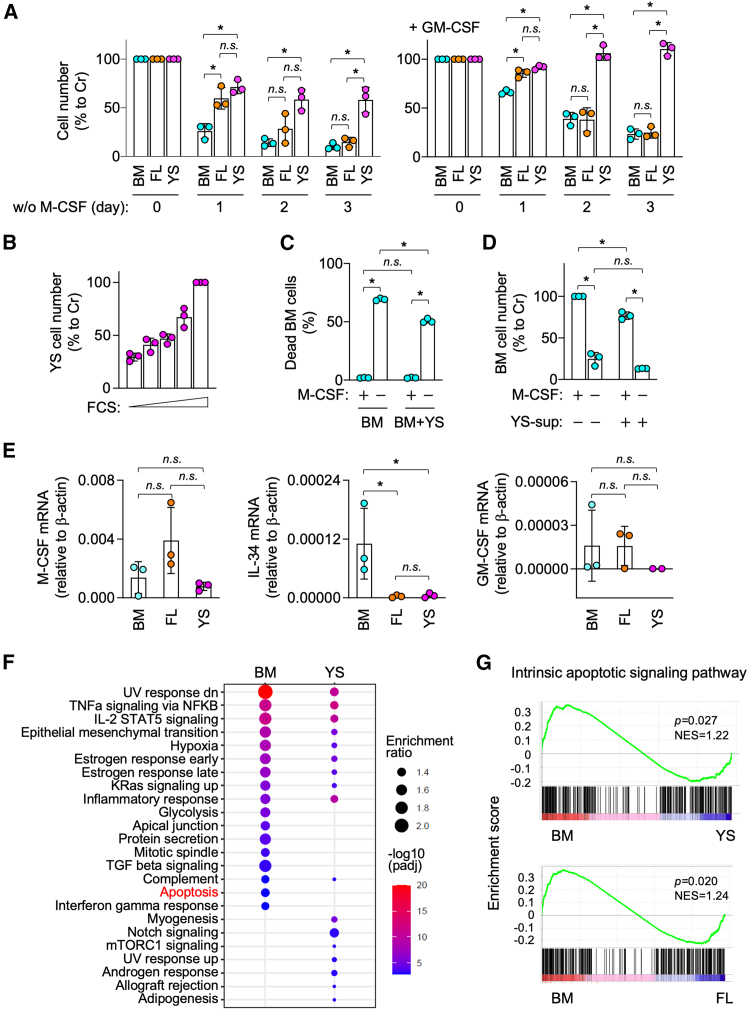
Survival of expanded macrophages under M-CSF-free culture (A) BM, FL, or YS lines (*n* = 3 for each group) were seeded without (w/o) M-CSF (left), or without M-CSF but with GM-CSF (right), cultured for 1, 2 or 3 days, and analyzed for their survival by the MTT assay. The survival level shown is the percentage to that of seeded cells (day 0) (mean ± SD). *n.s.*, not significant. ^∗^*p* < 0.05. [two-way ANOVA with Tukey’s multiple comparisons test]. (B) YS lines (*n* = 3) were seeded without M-CSF, cultured with different concentrations of fetal calf serum (FCS; 0, 0.3, 1, 3, or 10%) for 4 h, and analyzed for their survival by the MTT assay (mean ± SD). The level shown is the percentage to that of 10% FCS-containing culture. (C) Carboxyfluorescein succinimidyl ester-labeled BM line #1 was cultured in the presence or absence of M-CSF, or co-cultured with YS line #1 (1:5, “BM + YS”) in the presence or absence of M-CSF, and cultured for 24 h. The percentage of dead BM line #1 was quantified by flow cytometry (mean ± SD). *n.s.*, not significant. ^∗^*p* < 0.05. [two-way ANOVA with Sidak’s multiple comparisons test]. (D) BM line #1 was cultured in the presence or absence of M-CSF for 24 h, or seeded with media containing the supernatants collected from YS line #1 (50% v/v, "YS-sup"), and cultured in the presence or absence of M-CSF for 24 h. The YS-sup was prepared by culturing with M-CSF-free media for 3 days. The survival of BM line #1 was assessed by the MTT assay (mean ± SD). The level shown is the percentage to that of the M-CSF-containing/YS-sup-free culture. *n.s.*, not significant. ^∗^*p* < 0.05. [two-way ANOVA with Tukey’s multiple comparisons test]. (E) BM, FL, or YS lines (*n* = 3 for each group) were analyzed for the expression of M-CSF mRNA (left), IL-34 mRNA (middle), or GM-CSF mRNA (right) by qRT-PCR. The expression level shown is relative to that of β-actin mRNA (mean ± SD). *n.s.*, not significant. ^∗^*p* < 0.05. [One-way ANOVA]. (F) BM and YS lines (*n* = 2 for each group) were analyzed for the most significant pathways in the open chromatin regions using ATAC-seq data. In the dot plot, the size and color represent the enrichment ratio and -log10 (adjusted *p*-value, padj), respectively. (G) BM, FL, or YS lines (*n* = 3 for each group) were subjected to the gene set enrichment analysis of intrinsic apoptotic signaling pathway using RNA-seq data. The normalized enrichment score (NES) and *p* value are summarized. BM versus YS lines and BM versus FL lines are shown in upper and lower panels, respectively. See also Figure S9.

We next examined how expanded macrophages, in particular, YS lines, could resist cell death by M-CSF-depletion. Among three groups, YS lines survived without M-CSF most strongly and for the longest period (Figure 6A, left panel). FL lines survived more strongly than BM lines on day 1, but not on day 2 or day 3 (Figure 6A, left panel). We previously demonstrated that GM-CSF supported the survival of BM lines.^17^ GM-CSF also supported the survival of FL lines, and slightly induced the proliferation of YS lines without M-CSF (Figure 6A, right panel), further confirming the high proliferative capacity and resistance to cell death of YS lines. Components in fetal calf serum (FCS) might allow such M-CSF-free longer survival of YS lines as they severely died during 4 h-culture with lower concentrations of FCS (Figure 6B).

The co-culture with YS lines did not potentiate the M-CSF-free survival of BM lines (Figure 6C). The addition of the supernatants collected from YS lines also failed to markedly potentiate the M-CSF-free survival of BM lines (Figure 6D). The mRNA expression level of M-CSF, IL-34 (an alternative ligand of M-CSF receptor^24^), or GM-CSF was not necessarily high in YS lines (Figure 6E). Among the three cytokines, M-CSF showed a relatively high mRNA expression level (Figure 6E). However, when assessed by ELISA, the concentration of M-CSF in media conditioned by YS lines was <10 pg/m, the level of which was lower than that required to support the proliferation or survival of YS lines (>1 ng/mL). These results did not support an autocrine proliferation or survival in YS lines. Meanwhile, the apoptosis pathway was one of the most significant pathways in open chromatin regions in BM lines even when they were maintained in the presence of M-CSF (Figures 6F and S9). In addition, genes involved in intrinsic apoptotic signaling pathway were enriched in BM lines even when they were maintained in the presence of M-CSF (Figure 6G). Thus, it appeared that the relatively longer M-CSF-free survival of YS lines was due to differences in the intrinsic gene expression profile and chromatin accessibility among BM, FL, and YS lines.

### YS lines proliferate when transplanted into mice

**Figure 7:**
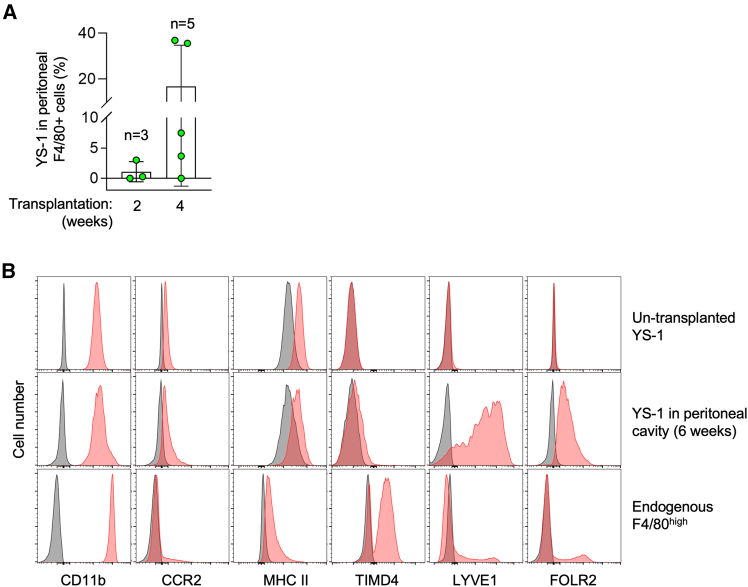
Proliferation and cell surface marker expression of expanded yolk sac macrophages after transplantation into mice (A) ZsGreen-expressing YS line #1 was transplanted into the peritoneal cavity of 8-week-old male mice. Two (*n* = 3) or four weeks (*n* = 5) after the transplantation, the percentage of the ZsGreen-expressing YS line #1 in the peritoneal F4/80-positive macrophages was assessed by flow cytometry (mean ± SD). (B) ZsGreen-expressing YS line #1 was transplanted into the peritoneal cavity of 5 or 6-week-old male mice. Six weeks after the transplantation, the peritoneal F4/80^high^ ZsGreen-positive cells were analyzed for the expression of CD11b, CCR2, MHC II (I-A/I-E), TIMD4, LYVE1, or FOLR2 by flow cytometry (middle). The peritoneal cells collected from six mice were combined. The un-transplanted control YS line #1 (top) or endogenous F4/80^high^ peritoneal macrophages (bottom) were analyzed as a reference. See also Figure S10.

In this study, we also performed a transplantation experiment using YS lines as an example. When transplanted into the peritoneal cavity of C57BL/6 mice, the percentage of YS line #1 in the peritoneal F4/80-positive macrophages was quite low 2 weeks after the transplantation, but increased in several mice 4 weeks after the transplantation (Figure 7A). The result suggested that YS line #1 could proliferate in mice, as observed in culture.

As mentioned earlier, TIMD4, LYVE1, and FOLR2 were recently identified as the core cell surface markers of embryo-derived macrophages.^23^ However, YS line #1 almost completely lost the expression of these markers, presumably during the long-term *ex vivo* culture (Figure 7B, top panels; Figure S10). However, once again, when transplanted into mice and analyzed 6 weeks later, YS line #1, which were detected within F4/80^high^ large peritoneal macrophage subpopulation (Figure S10), expressed TIMD4 at a detectable level (Figure 7B, middle panel), although weakly when compared to the endogenous subpopulation (Figure 7B, bottom panel). In contrast to TIMD4, LYVE1, and FOLR2 were expressed in a fraction of peritoneal macrophages.^26^^,^^27^^,^^28^ The level of LYVE1 or FOLR2 of the transplanted YS #1 was comparable to that of the LYVE-positive or FOLR2-positive F4/80^high^ endogenous macrophages (Figure 7B, middle and bottom panels). Such altered expression in YS line #1 after the transplantation was not observed for other cell surface markers, such as CD11b, CCR2, and MHC II (I-A/I-E) (Figure 7B, top and middle panels). The result suggests that when transferred into the peritoneal cavity of mice, YS lines could proliferate and regain the expression of several original cell surface markers, but not the expression patterns of endogenous F4/80^high^ peritoneal macrophages.

## Discussion

In this study, we established genetically unmodified but stably proliferating macrophage lines from YS, FL, and BM under the same conditions, and compared their features under the same setting. In support of the idea that embryonic macrophages persist in many adult tissues due to their higher proliferative capacity, the expanded embryonic macrophages, in particular, YS lines proliferated faster than FL and BM lines (Figure 4). Interestingly, YS lines survived for a longer period than BM or FL lines under M-CSF-free culture (Figure 6), and YS and FL lines were more resistant to apoptotic cell death induced by staurosporine or nutlin-3 than BM lines (Figure 5). Thus, the present study strongly suggests that not only the higher proliferative capacity but also the higher survival or anti-apoptotic capacity enables embryonic macrophages to reside in tissues far longer than originally thought.

The present study also suggests that the long-term culture with repeated passages does not necessarily erase all the intrinsic features of macrophages because YS, FL, and BM lines were overlapped but not identical in phenotypes including the response to LPS (Figure 1C), gene expression profile (Figure 2), and chromatin accessibility (Figure 3). However, the fact remains that when cultured, cells lose part of their original gene expression signature, as exemplified by microglia.^29^^,^^30^ In fact, YS lines almost completely lost the expression of the core YS macrophage markers,^23^ TIMD4, LYVE1, and FOLR2 (Figure 7). However, it should be mentioned that when transplanted into mice, YS line gained the expression of these markers, although the recovery of TIMD4 was weak (Figure 7). Thus, it is likely that the adaptation to culture environment is reversible at least for macrophages expanded from YS.

In addition to MafB/c-Maf-deficient macrophages^31^^,^^32^^,^^33^ and alveolar macrophages,^32^^,^^34^ macrophages originating from FL^9^ and BM^14^^,^^17^ have been demonstrated to proliferate stably and unlimitedly in culture. To the best of our knowledge, the present study established, for the first time, stably proliferating macrophage lines from YS. Tissue-resident macrophages originating from YS or FL macrophages are developmentally distinct from macrophages derived from BM. Consistent with this, FL and YS lines are relatively close but distant from BM lines in the gene expression profile and chromatin accessibility (Figures 2 and 3). The recent finding that not only macrophages derived from BM but also those originating from YS or FL are present in many adult tissues.^3^^,^^4^^,^^5^ However, it remains unclear what is the physiological or pathological role of each macrophage population. The YS, FL and BM lines established in this study are helpful to answer the fundamental question. Previously, van de Laar et al. collected F4/80^int^CD11b^hi^Ly6C^hi^ monocytes from adult BM, F4/80^int^CD11b^hi^Ly6C^hi^ monocytes from E15.5 FL and F4/80^hi^CD11b^lo^Ly6C^lo^ macrophages from E12.5 YS, and analyzed their gene expression profiles without *ex vivo* expansion.^8^ In their microarray analysis, genes involved in antigen processing and presentation were slightly enriched in BM monocytes, genes involved in regulation of endocytosis were slightly enriched in YS macrophages, and genes involved in response to virus were enriched in both BM monocytes and YS macrophages.^8^ Such differences were not necessarily detected in our RNA-seq analysis using BM, FL, and YS lines (Figure S11). For example, genes involved in response to virus were not enriched in BM lines (Figure S11, bottom panels). Nevertheless, cell proliferation-associated genes were enriched in unexpanded YS macrophages/FL monocytes^8^ and expanded YS/FL lines (Figure 4D). Thus, the YS, FL, and BM lines established in the present study would be useful to clarify molecular mechanism by which macrophages derived from BM, FL, or YS have different proliferative capacity.

In this study, we used M-CSF as the growth factor for macrophages because it is required for macrophage development in many tissues.^15^^,^^16^ Meanwhile, GM-CSF is generally used for the culture of alveolar macrophages^32^^,^^34^ because it is critical for their functions.^35^ When we established FL lines using GM-CSF, their proliferation was comparable to that of FL lines established using M-CSF (will be published elsewhere). Thus, both cytokines can be used for the establishment of stably proliferating macrophage lines, although lines once established using M-CSF lose the efficient proliferative response to GM-CSF (Figure 6A, right panel), and vice versa (will be published elsewhere).

YS, FL, and BM lines are ready-to-use macrophages, although their differentiation status is not clearly defined. They can reduce the repeated use of mice, including pregnant mice to prepare YS or FL macrophages. They would be also useful for a wide range of research fields, including the interaction with pathogens, innate immunity, and tumor-associated macrophages, and chimeric antigen receptor (CAR) macrophages. The present study indicates that macrophages of BM, FL, and YS can proliferate *ex vivo* and do not lose their core intrinsic features even after unlimited expansion, providing new models to characterize macrophages with different developmental origins.

### Limitations of the study

BM, FL, and YS lines established in the present study using M-CSF appeared to show a weak response to GM-CSF (Figure 6A). For example, GM-CSF supported the proliferation of primary BM-derived macrophages (Figure S12A), but not of BM lines (Figure S12B). Nevertheless, GM-CSF could support the survival of BM lines, albeit weakly (Figure S12B). This weak response of BM lines to GM-CSF is presumably due to their adaptation to M-CSF during the long-term culture with M-CSF, but the precise underlying mechanism remains unexplored.

The percentage of YS line #1 in peritoneal F4/80^+^ cells was highly variable among mice when assessed 4 weeks after the transplantation (Figure 7A). The similar result was obtained even when we repeated the experiments under similar conditions (Figure S13), although the reason for the variability among transplanted mice remains unclear. In Figures 7 and S13, we used male mice as the recipients. Thus, it may be necessary to test whether the similar result is obtained when we repeat the experiment using female mice. It is also necessary to clarify why the transplanted YS lines cannot regain the expression patterns of endogenous F4/80^high^ peritoneal macrophages.

In this study, we transplanted macrophages expanded from YS into peritoneal cavity of mice. In the next study, it will be important to test the ability of YS lines to colonize brains as transcriptionally and functionally resident microglia-like cells, because microglia are well known to develop from YS progenitor cells.^4^^,^^5^

## Resource availability

### Lead contact

Requests for further information and resources should be directed to and will be fulfilled by the lead contact, Shinya Suzu (ssuzu06@kumamoto-u.ac.jp).

### Materials availability

All unique/stable reagents generated in this study are available from the lead contact with a completed materials transfer agreement.

### Data and code availability

• The RNA-seq and ATAC-seq data have been deposited in the National Center for Biotechnology Information Gene Expression Omnibus as GSE276196 and GSE276197, respectively.
• This paper does not report original code.
• Any additional information required to reanalyze the data reported in this paper is available from the lead contact upon request.

## Acknowledgments

We thank Jane Doe of the Liaison Laboratory Research Promotion Center of Kumamoto University for technical support. We also thank K. Nasu and I. Suzu for the technical and secretarial assistance, respectively. This study was supported by a grant from the 10.13039/501100001691Japan Society for the Promotion of Science (JSPS) (22K08456 to N.T.), and grants from the 10.13039/100017682Japanese Society of Hematology (to N.T. and S.S.). This work was also supported by MEXT Promotion of Development of a Joint Usage/ Research System Project: Coalition of Universities for Research Excellence Program (JPMXP1323015486).

## Author contributions

Conceptualization, S.S.; methodology, S.A.H.; software, K.E.; investigation, S.A.H., N.T., Y.M.E., R.A.A., H.O.-A., and S.U.; resources, S.O., H.T., S.M.-K., and M.O.; writing – original draft, S.A.H. and S.S.; writing – review and editing, S.A.H., N.T., Y.M.E., R.A.A., S.U., K.E., S.H., M.O., and S.S.; funding acquisition, N.T. and S.S.

## Declaration of interests

The authors declare no competing interests.

## STAR★Methods

### Key resources table

**Table:** 

REAGENT or RESOURCE	SOURCE	IDENTIFIER
**Antibodies**		

Anti-M-CSF receptor	Cell Signaling Technology	#3152; RRID: AB_2085233
Anti-pTyr809 M-CSF receptor	Cell Signaling Technology	#3154; RRID: AB_2085231
Anti-cleaved caspase-3	Cell Signaling Technology	5A1E; RRID: AB_2070042
Anti-PARP	Cell Signaling Technology	#9542; RRID: AB_2160739
Anti-β-actin	Abcam	EPR16769; RRID: AB_2737344
APC anti-CD11b	BioLegend	M1/70; RRID: AB_312794
FITC anti-CD11b	BioLegend	M1/70; RRID: AB_312788
FITC anti-F4/80	BioLegend	BM8; RRID: AB_893500
APC/Cyanine7 anti-F4/80	BioLegend	BM8; RRID: AB_893489
APC anti-M-CSF receptor	BioLegend	AFS98; RRID: AB_2085222
FITC anti-H-2	BioLegend	M1/42; RRID: AB_1236475
Pacific Blue anti-I-A/I-E	BioLegend	M5/114.15.2; RRID: AB_493528
APC anti-CCR2	BioLegend	SA203G11; RRID: AB_2810414
PerCP/Cyanine5.5 anti-TIMD4	BioLegend	RMT4-54; RRID: AB_2876458
APC anti-FOLR2	BioLegend	10/FR2; RRID: AB_2721312
PE-cyanine7 anti-LYVE1	Thermo Fisher Scientific	ALY7; RRID: AB_2802237
PE anti-TNF-α	BioLegend	MP6-XT22; RRID: AB_315428

**Chemicals, peptides, and recombinant proteins**		

Recombinant M-CSF	Morinaga Milk Industry	N/A
Recombinant IFN-γ	BioLegend	#575304
Recombinant GM-CSF	Miltenyi Biotec	#130-095-739
LPS	Alexis	E. coli 0111: B4
Staurosporine	Selleckchem	S1421
Nutlin-3	Selleckchem	S1061

**Oligonucleotides**		

PCR primers (iNOS): 5′-ACCTTGTTCAGCTACGCCTT-3′ 5′- CATTCCCAAATGTGCTTGTC-3'	Eurofins	N/A
PCR primers (M-CSF): 5′-GCCACATTGATTGGGAATGGAC-3′ 5′-TCAAAGGCAATCTGGCATGAAG-3′	TaKaRa-Bio	N/A
PCR primers (IL-34): 5′-CAGTACAAGAACCGGCT TCAGTACA-3′ 5′-CCGAAGCTCTCGCTCACTCA-3′	TaKaRa-Bio	N/A
PCR primers (GM-CSF): 5′-AAGGGCGCCTTGAACATGA-3′ 5′-AAATCCGCATAGGTGGTAACTTGTG-3′	TaKaRa-Bio	N/A
PCR primers (β-actin): 5′-CATCCGTAAAGACCTCTATGCCAAC-3′ 5′-ATGGAGCCACCGATCCACA-3′	TaKaRa-Bio	N/A

**Deposited data**		

RNA-Seq	NCBI GEO	GSE276196
ATAC-Seq	NCBI GEO	GSE276197

**Software and algorithms**		

Prism 10	GraphPad	version 10.0
ImageJ	NIH	1.52n
FlowJo	FlowJo LLC	version 10.0
iDEP (v0.96)	South Dakota State Univ.	Ge et al.^36^
RNAseqChef	Etoh and Nakao	Etoh and Nakao^37^
Limma	Ritchie et al.	Ritchie et al.^38^
rGREAT	Gu and Hübschmann	Gu and Hübschmann^39^
HOMER	Heinz et al.	Heinz et al.^40^
ComplexHeatmap	Gu et al.	Gu et al.^41^

### Experimental model and study participant details

#### Establishment of BM, FL, and YS lines

C57BL/6 mice were used to establish M-CSF-dependent FL or YS lines, as described previously for M-CSF-dependent BM lines.^17^ Total cells were collected from bone marrow (adult), fetal liver (E14.5) or yolk sac (E9.5). Yolk sac was dispersed using forceps and scissors followed by repeated pipetting, and cell suspension was prepared. To obtain an enough number of cells for the initial culture, cells pooled from two or more mice were used. Those cells were suspended into RPMI1640 media supplemented with 10% FCS and 100 ng/mL recombinant human (rh)M-CSF (complete media). When proliferated, cells were subjected to repeated detachment/reseeding. Total bone marrow cells (1x10^6^ cells/mL) or total fetal liver cells (5x10^5^ cells/mL) were initially seeded onto 10-cm non-coated polystyrene dishes (10 mL media/dish, >3 dishes), and cultured for 5-6 days. The adherent cells with approximately 80-90% confluency were detached by incubating with 2 mL 0.25% trypsin containing 1 mM EDTA (Fuji Film) for 3 min at 37°C, and combined with floating cells. Those cells were re-suspended at 3-5x10^5^ cells/mL, seeded onto 10-cm dishes, and cultured for 5-10 days. In the fourth passage (bone marrow) or third passage (fetal liver), most adherent cells ceased to proliferate and began to die. Instead, several growing colonies composed of macrophage-like cells appeared approximately two months after the initial cell seeding. When these colonies were collected and cultured for additional two months (bone marrow) or one month (fetal liver) with the detachment/reseeding every 7-10 days, their proliferation speed became relatively stable. Total yolk sac cells (2x10^5^ cells/mL) were initially seeded onto 24-well non-coated polystyrene plates (500 μL/well, >3 wells), and cultured for 7 days. The adherent cells with approximately 80-90% confluency were detached as above, and combined with floating cells. Those cells were re-suspended at approximately 1x10^5^ cells/mL, seeded onto 6-well non-coated polystyrene plates, and cultured for 5-10 days. In the third passage, the adherent cells did not necessarily cease to proliferate, but many growing colonies composed of macrophage-like cells appeared approximately one and a half months after the initial cell seeding. When these colonies were collected and cultured for additional one month with the detachment/reseeding every week, their proliferation speed became relatively stable. The bulk culture obtained from a separate dish or well was defined as an independent line. Mice used in this study were maintained under specific pathogen-free conditions at the animal research facility of Kumamoto University. Procedures and protocols for animal experiments were approved by the Institutional Animal Care and Use Committee of Kumamoto University (A2023-099, April 1, 2023).

### Method details

#### RNA-Seq

Total RNA was isolated using ISOGEN II (Nippon Gene, Tokyo, Japan). RNA libraries were prepared using Next Poly(A) mRNA Magnetic Isolation Module and Ultra II Directional RNA Library Prep Kit for Illumina (New England Biolabs) and were sequenced on NextSeq 500 (Illumina) in single read mode with the read length of 75 nt. iDEP (v0.96)^36^ was used to generate normalized gene expression of raw read counts using EdgeR:log2 (CPM+c) method, determine differentially expressed genes using DESeq2,^42^ and generate *k*-means clustering. Volcano plots were visualized using RNAseqChef.^37^ Gene set enrichment analysis (GSEA)^43^ was done using the GSEA software (v4.0.3) with the default parameters. Pathways with NOM P < 0.05 were considered to be significantly enriched. The data have been deposited in the National Center for Biotechnology Information Gene Expression Omnibus (GSE276196).

#### ATAC-Seq

DNA samples for ATAC-Seq were prepared using ATAC-Seq Kit (Active Motif).^44^^,^^45^ Purified DNAs were sequenced on NextSeq 500 using NextSeq 500/550 High Output v2 Kit (Illumina) to obtain single end 75 nt reads. A count matrix of the coverage in each reproducible peak-called genomic locus was created from coverage data (bigwig files) and bed files. Differentially accessible regions were detected using the limma package^38^ with the following thresholds: fold change > 2 and false discovery rate < 0.1. Functional enrichment analysis was performed using the rGREAT package.^39^ Motif enrichment analysis was performed using hypergeometric optimization of motif enrichment (HOMER).^40^ The *k*-means clustering analysis was performed using the ComplexHeatmap package.^41^ The data have been deposited in the National Center for Biotechnology Information Gene Expression Omnibus (GSE276197).

#### Cytokines and reagents

rhM-CSF was provided by Morinaga Milk Industry (Kanagawa, Japan), and added to the culture at a final concentration of 100 ng/mL. Recombinant mouse (rm)IFN-γ and rmGM-CSF were purchased from BioLegend and Miltenyi Biotec, respectively, and added to the culture at a final concentration of 10 ng/mL. LPS of *Escherichia coli* serotype 0111:B4 was purchased from Alexis, and added to the culture at a final concentration of 10 or 100 ng/mL. Staurosporine and nutlin-3 were purchased from Selleckchem, dissolved in DMSO, and added to the culture at a final concentration of 1 μM (staurosporine) or 10 μM (nutlin-3). The same volume of DMSO was added as a vehicle control.

#### Flow cytometry

##### Cell surface

The cell surface expression of CD11b, F4/80, M-CSF receptor, MHC I (H-2), MHC II (I-A/I-E), CCR2, TIMD4, LYVE1, or FOLR2 was analyzed by flow cytometry. In brief, cells were detached using enzyme-free cell dissociation buffer (Millipore), stained with fluorescent dye-labeled antibodies, and analyzed on Cytek Northern Lights (Cytek Bioscience) using FlowJo software (FlowJo LLC). The antibodies used were as follows: FITC-labeled or allophycocyanin (APC)-labeled anti-CD11b (M1/70), FITC-labeled or APC/Cyanine7-labeled anti-F4/80 (BM8), APC-labeled anti-M-CSF receptor (AFS98), FITC-labeled anti-H-2 (M1/42), Pacific Blue-labeled anti-I-A/I-E (M5/114.15.2), APC-labeled anti-CCR2 (SA203G11), PerCP/Cyanine5.5-labeled anti-TIMD4 (RMT4-54), APC-labeled anti-FOLR2 (10/FR2) (all from BioLegend), and PE-cyanine7-labeled anti-LYVE1 (ALY7; Thermo Fisher Scientific).

##### Intracellular

Cells were stimulated with LPS or IFN-γ, and their intracellular level of TNF-α was analyzed by flow cytometry.^46^ In brief, cells were incubated with monensin during the last 4 hours of stimulation, detached, fixed, permeabilized, and stained with PE-labeled anti-TNF-α (MP6-XT22; BioLegend).

##### Phagocytosis

Phagocytic activity was also analyzed by flow cytometry as described previously.^47^ Prior to analysis, cells were incubated with fluorescent microspheres (fluoresbrite carboxylate microspheres with a 0.7 μm diameter; Polysciences) for 2 hours.

##### Apoptosis

Cells were M-CSF-depleted or treated with staurosporine or nutlin-3. Then, apoptotic cells were detected by flow cytometry,^17^ using PE Annexin V apoptosis detection kit with 7-AAD (BioLegend).

##### Cell death

In a selected experiment (Figure 6C), BM line #1 was labeled with carboxyfluorescein succinimidyl ester (CFSE) and co-cultured with YS line #1. Then, the percentage of dead BM #1 line was quantified by flow cytometry, using LIVE/DEAD Aqua (Thermo Fisher Scientific).

#### Cell count

The viable cells were enumerated by trypan blue dye exclusion method. The number of cells was also assessed using the MTT reagent. The absorbance of the wells was measured at 590 nm.

#### Western blotting

Western blotting was performed as described previously.^47^ The antibodies used were as follows: anti-M-CSF receptor (#3152; Cell Signaling Technology), anti-pTyr809 M-CSF receptor (#3154; Cell Signaling Technology), anti-cleaved caspase-3 (5A1E; Cell Signaling Technology), anti-PARP (#9542; Cell Signaling Technology), and anti-β-actin (EPR16769; Abcam). Detection was performed using HRP-labeled secondary antibodies (GE Healthcare), western blot ultra-sensitive HRP substrate (TaKaRa-Bio), and ImageQuant LAS4000 image analyzer (GE Healthcare).

#### qRT-PCR

The expression of iNOS, M-CSF, IL-34, or GM-CSF was analyzed by real-time RT-PCR as described previously.^47^ RNA was isolated, and cDNA was prepared using M-MLV RT (Invitrogen). Real-time RCR was performed using SYBR Premix Ex Taq II (TaKaRa-Bio) and LightCycler (Roche). β-actin mRNA was quantified as an internal control. The levels of mRNA expression were calculated using the ΔΔCt method. The primers used are as follows: 5′-ACCTTGTTCAGCTACGCCTT-3′ and 5'- CATTCCCAAATGTGCTTGTC-3' (iNOS), 5′-GCCACATTGATTGGGAATGGAC-3' and 5′-TCAAAGGCAATCTGGCATGAAG-3' (M-CSF), 5′-CAGTACAAGAACCGGCTTCAGTACA-3' and 5′-CCGAAGCTCTCGCTCACTCA-3' (IL-34), 5′-AAGGGCGCCTTGAACATGA-3' and 5′-AAATCCGCATAGGTGGTAACTTGTG-3' (GM-CSF), and 5′-CATCCGTAAAGACCTCTATGCCAAC-3' and 5′-ATGGAGCCACCGATCCACA-3' (β-actin).

#### Transplantation into mice

YS line #1 was engineered to express ZsGreen by the lentiviral infection system. The lentiviruses were prepared by co-transfecting pLVSIN-EF1α-IRES-ZsGreen1 vector and Lentiviral High Titer Packaging mix (both from TaKaRa-Bio) into Lenti-X-293T cells (Clontech) using TransIT-293 Transfection reagent (Mirus Bio), and concentrated using Lenti-X concentrator (Clontech). The ZsGreen-expressing YS line #1 was enriched by cell sorting using FACSAria II (BD Biosciences), and transplanted into the peritoneal cavity of C57BL/6 male mice (1x10^7^ cells/mouse). Then, peritoneal cells were collected after the lavage and analyzed by flow cytometry as described above.

### Quantification and statistical analysis

The density of bands in western blotting was quantified using the ImageJ software. Differences between multiple groups were analyzed by a one-way or two-way ANOVA with Tukey’s or Sidak’s multiple comparisons test. All statistical analyses were conducted using PRISM 10 (GraphPad). Statistical significance was set at *p* < 0.05.
