# Supplementary material for: Macrophages with different origins proliferate ex vivo and do not lose their core intrinsic features
Source: iScience. 2025 May 12;28(6):112635. doi: 10.1016/j.isci.2025.112635 (PMC12159446; doi:10.1016/j.isci.2025.112635)
Supplement: Document S1. Figures S1–S13 [file mmc1.pdf]

iScience, Volume *28*

Supplemental information

Macrophages with different origins proliferate *ex vivo* and do not lose their core intrinsic features

Sara A. Habash, Naofumi Takahashi, Youssef M. Eltalkhawy, Randa A. Abdelnaser, Hiromi Ogata-Aoki, Seiji Okada, Hitoshi Takizawa, Shingo Usuki, Kan Etoh, Shinjiro Hino, Saori Morino-Koga, Minetaro Ogawa, and Shinya Suzu

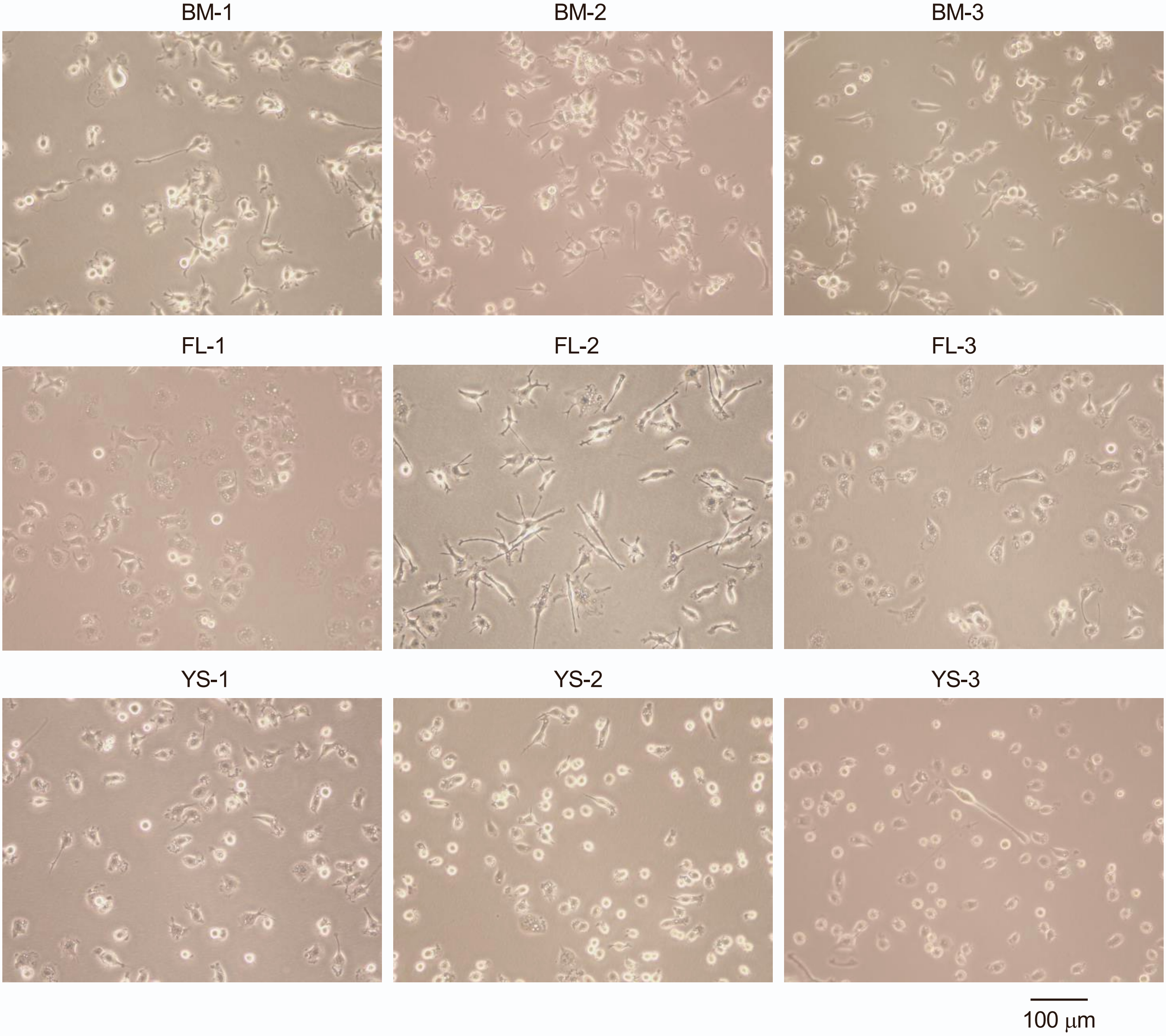

Figure S1. Morphology of expanded macrophages. The morphology of macrophage lines expanded from bone marrow (BM), fetal liver (FL), or yolk sac (YS) is shown (3 lines for each group). Scale bar: 100 µm.

Figure S1

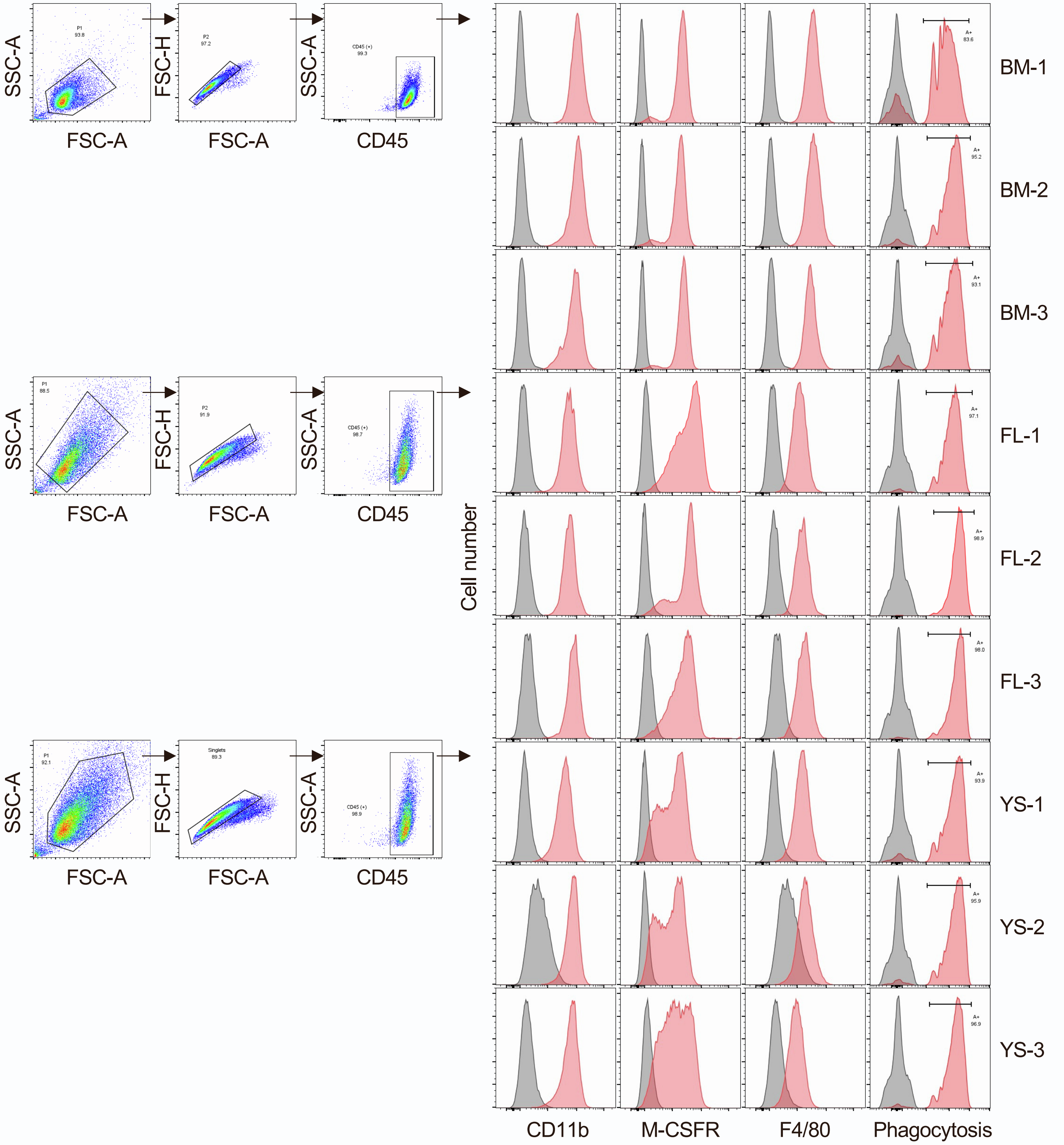

Figure S2. The gating and histograms of Figure 1A.

Figure S2

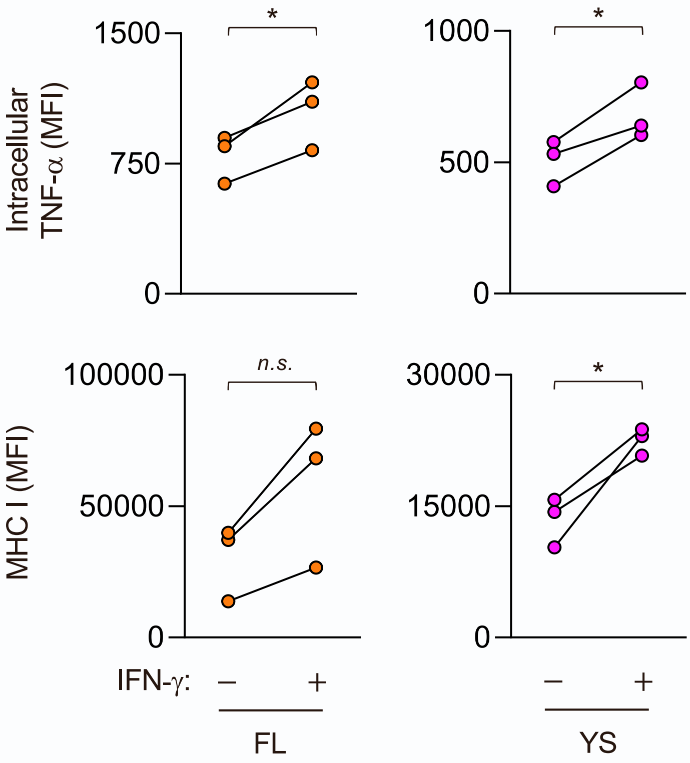

Figure S3. Response of FL and YS lines to IFN-γ.
(Related to Figure 1D)

FL or YS lines (n=3 for each group) were cultured in the absence or presence of 10 ng/mL IFN-γ for 24 hours, and analyzed for the intracellular level of TNF-α protein or cell surface level of MHC I (H-2) by flow cytometry. The mean fluorescence intensity (MFI) is shown. *n.s.*, not significant. $*p < 0.05$. [Paired Student's *t*-test]

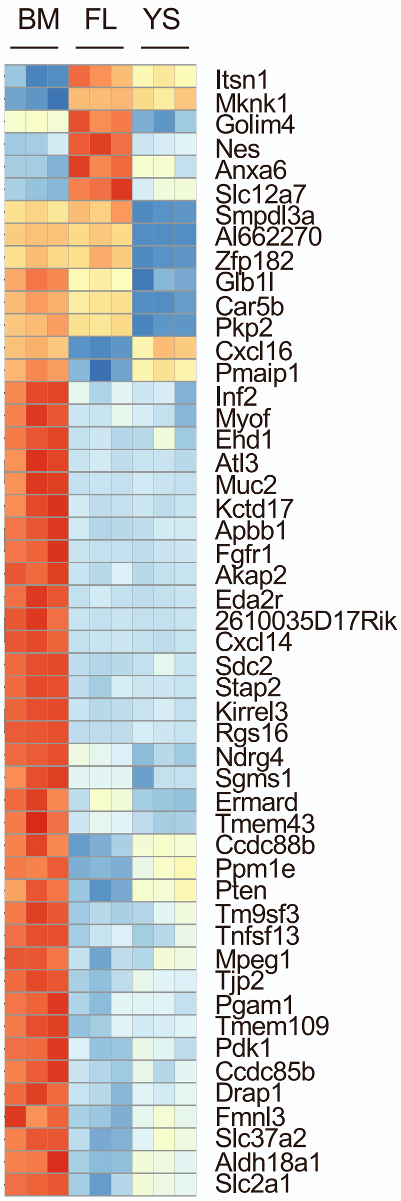

Figure S4. Top 50 differentially expressed genes among BM, FL, and YS lines. (Related to Figure 2)

BM, FL, or YS lines (n=3 for each group) were subjected to bulk RNA-Seq analysis. The top 50 differentially expressed genes among three groups are summarized in the heat map.

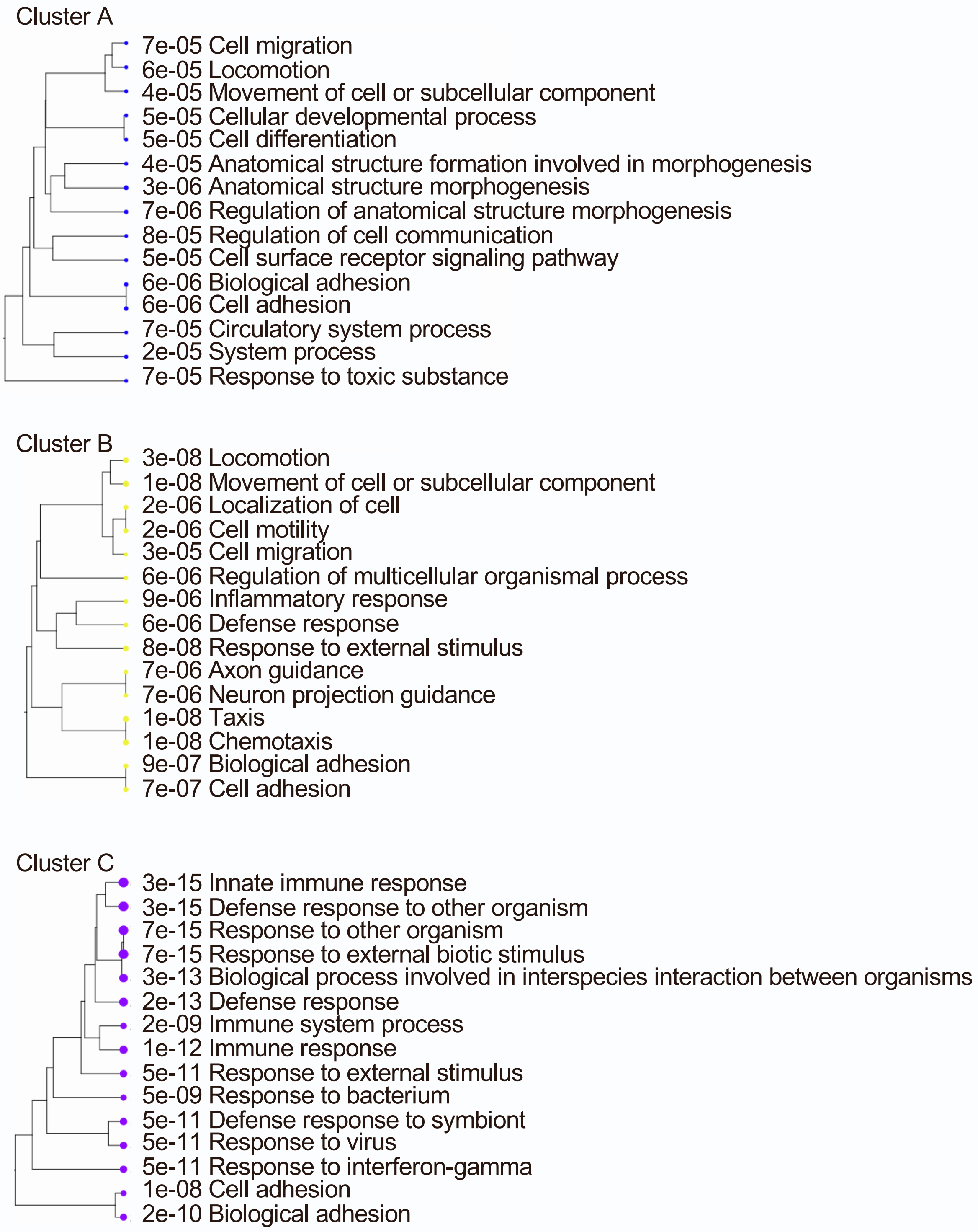

Figure S5. The gene ontology terms for biological process in Cluster A, B or C, in Figure 2C.

Figure S5

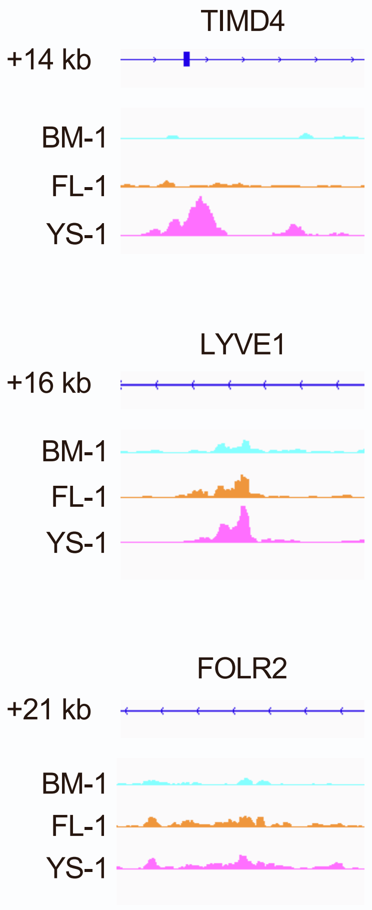

Figure S6. Integrative genomics viewer (IGV) tracks of TIMD4, LYVE1 and FOLR2.
(Related to Figure 3)
BM line #1, FL line #1, or YS line #1 was subjected to ATAC-Seq analysis. IGV tracks of TIMD4, LYVE1 and FOLR2 are shown.

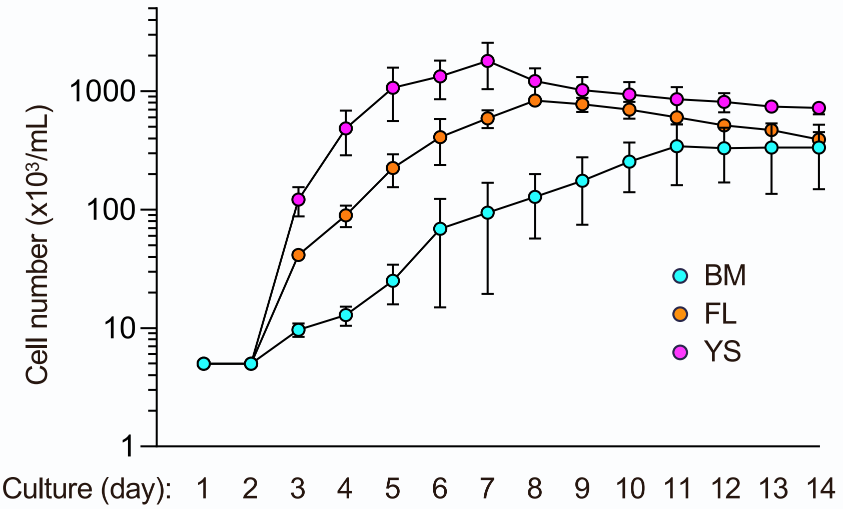

Figure S7. Proliferation of expanded macrophages. (Related to Figure 4A)

BM, FL, or YS lines (n=3 for each group) were seeded at $5x10^3$ cells/mL and cultured in the presence of 100 ng/mL M-CSF for up to day 14. The media were replaced every other day with fresh complete media containing M-CSF. The number of viable cells was counted by trypan blue dye exclusion method. Mean ± SD is shown.

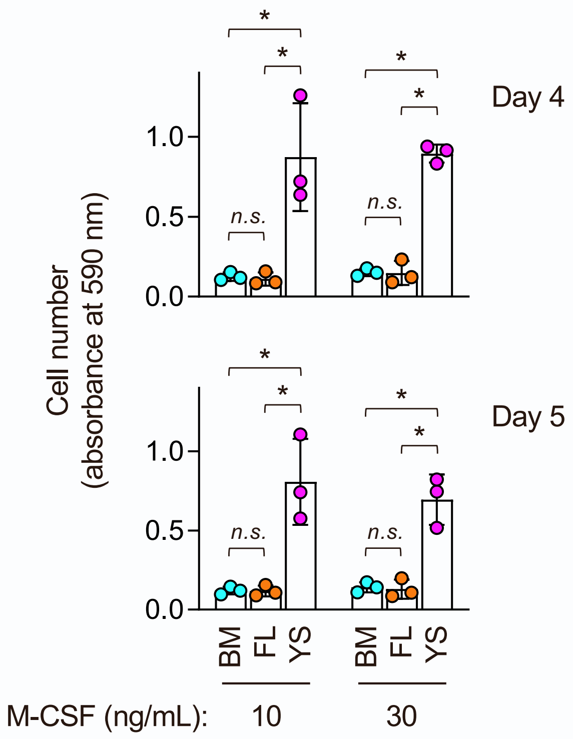

Figure S8. Proliferation of expanded macrophages.
(Related to Figure 4B)

BM, FL, or YS lines (n=3 for each group) were seeded at $1x10^5$ cells/mL, and cultured for 4 or 5 days in the presence of 10 or 30 ng/mL of M-CSF. The number of cells was monitored by the MTT assay, and the absorbance of the wells measured at 590 nm was shown. Mean ± SD is shown. *n.s.*, not significant. $*p < 0.05$. [Two-way ANOVA with Tukey's multiple comparisons test]

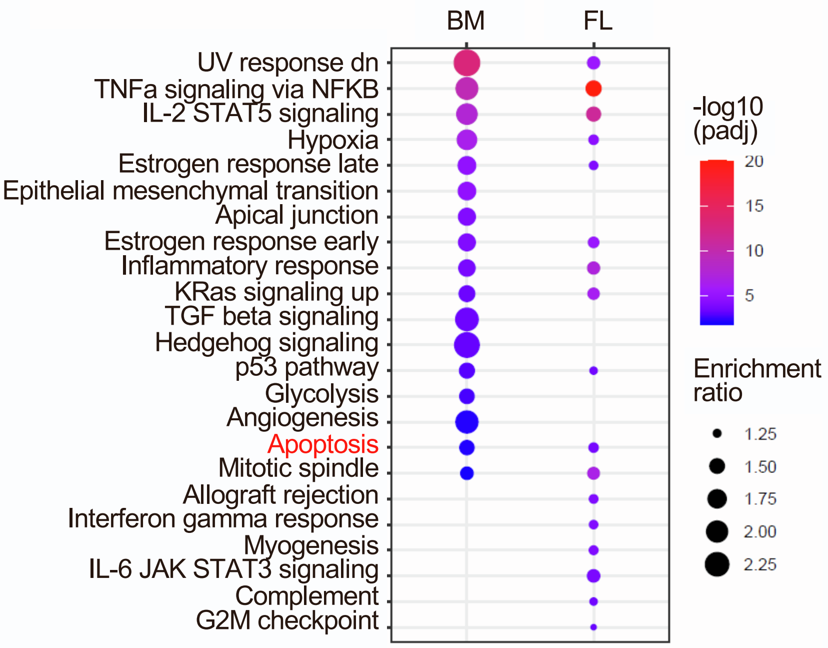

Figure S9. The most significant pathways in open chromatin regions between BM and FL lines.

(Related to Figure 6F)

BM and FL lines (n=2 for each group) were analyzed for the most significant pathways in the open chromatin regions using ATAC-Seq data. In the dot plot, the size and color represent the enrichment ratio and -log10 (adjusted *p*-value, padj), respectively.

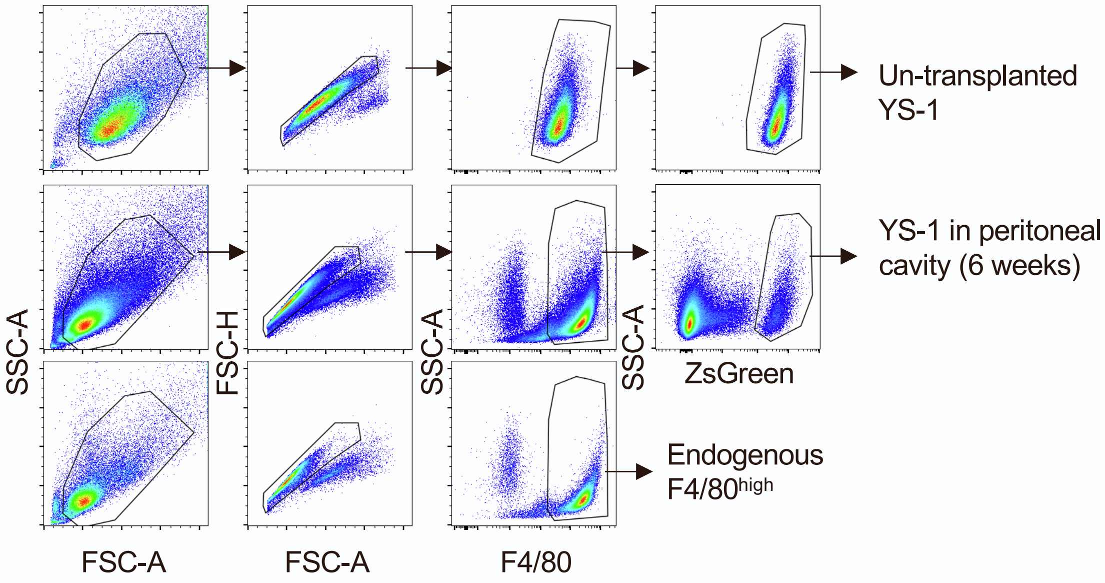

Figure S10. The gating of Figure 7B.

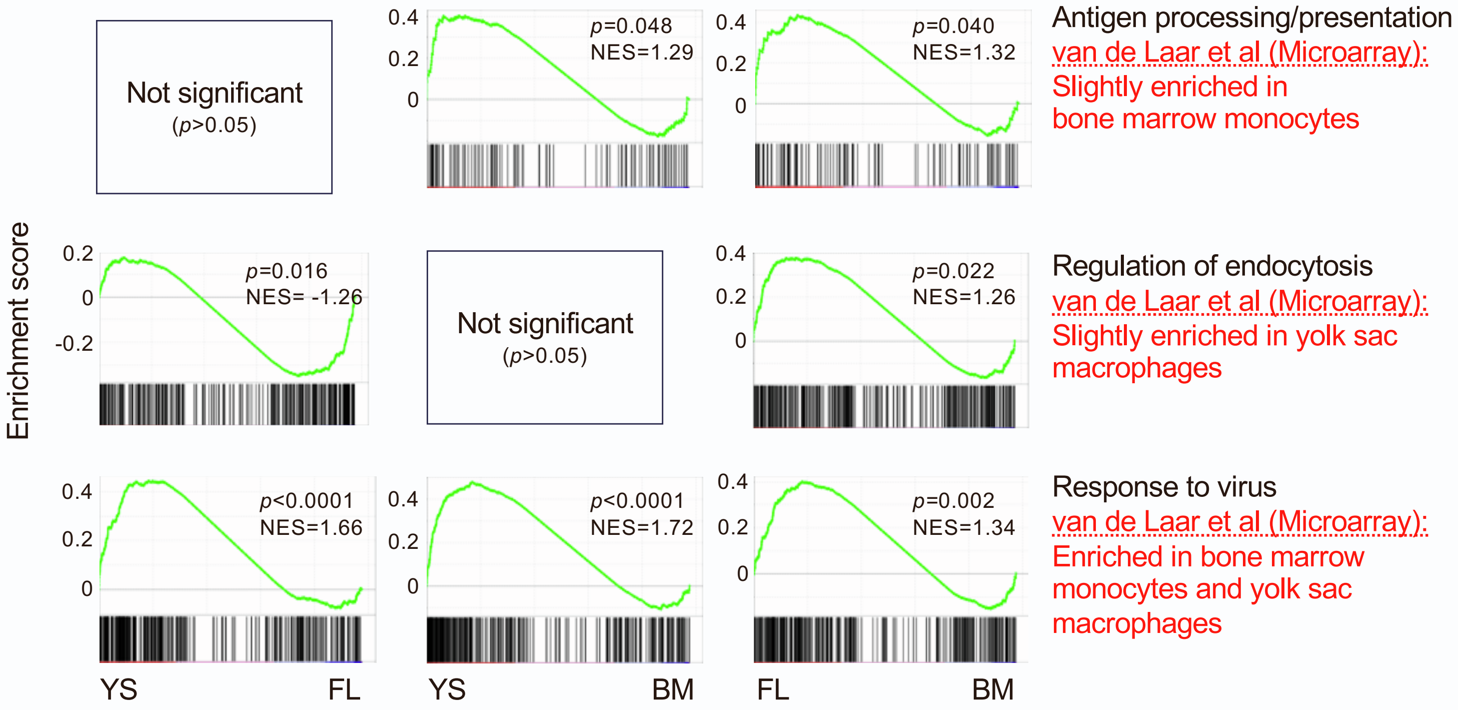

Figure S11. Gene set enrichment analysis (GSEA) using RNA-Seq data.
Using RNA-Seq data, BM, FL, or YS lines (n=3 for each group) were subjected to GSEA of the indicated gene ontology biological process (GO:BP) gene sets, which were identified as differentially enriched gene sets among unexpanded bone marrow monocytes, fetal liver monocytes and yolk sac macrophages by microarray (van de Laar et al.[8]) . The normalized enrichment score (NES) and *p* value are summarized. YS versus FL, YS versus BM, and FL versus BM are shown in left, middle, and right panels, respectively.

Figure S11

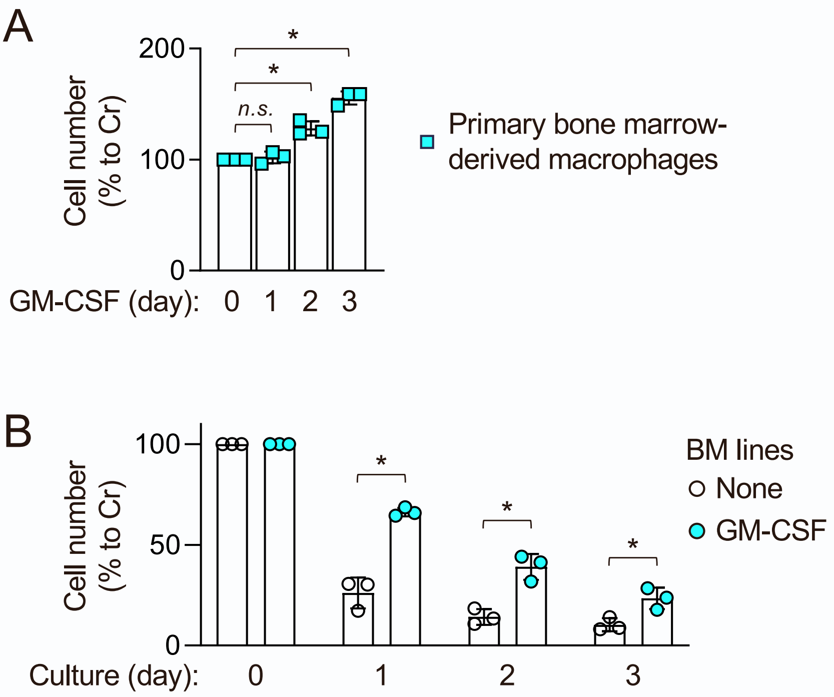

Figure S12. Effect of GM-CSF on proliferation/survival of primary bone marrow-derived macrophages and BM lines.

(Related to Figure 6A)

(**A**) The primary bone marrow-derived macrophages prepared using M-CSF were cultured in the presence of GM-CSF for 1, 2 or 3 days, and analyzed for their proliferation by the MTT assay. The proliferation level shown is the percentage to that of seeded cells (day 0). Mean ± SD is shown. *n.s.*, not significant. *$p < 0.05$. [One-way ANOVA with Dunnett's multiple comparisons test]

(**B**) BM lines (n=3) were seeded in the presence or absence of GM-CSF, cultured for 1, 2 or 3 days, and analyzed for their survival by the MTT assay. Mean ± SD is shown. The survival level shown is the percentage to that of seeded cells (day 0). *$p < 0.05$. [Two-way ANOVA with Sidak's multiple comparisons test]

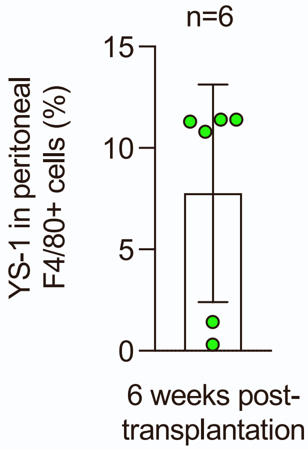

Figure S13. The percentage of YS #1 in peritoneal F4/80-positive macrophages.
(Related to Figure 7A)

ZsGreen-expressing YS line #1 was transplanted into the peritoneal cavity of 5 or 6-week-old male mice. Six weeks after the transplantation, the percentage of the ZsGreen-expressing YS line #1 in the peritoneal F4/80-positive macrophages was assessed by flow cytometry. Mean ± SD is shown.
